# Multicenter Cross-sectional Study on the Epidemiology of Human Metapneumovirus in Italy, 2022–2024, With a Focus on Adults Over 50 Years of Age

**DOI:** 10.1093/infdis/jiaf111

**Published:** 2025-07-16

**Authors:** Alessandro Mancon, Laura Pellegrinelli, Greta Romano, Elisa Vian, Valeria Biscaro, Giulia Piccirilli, Tiziana Lazzarotto, Sara Uceda Renteria, Annapaola Callegaro, Elisabetta Pagani, Elisa Masi, Guglielmo Ferrari, Cristina Galli, Francesca Centrone, Maria Chironna, Claudia Tiberio, Erasmo Falco, Valeria Micheli, Federica Novazzi, Nicasio Mancini, Tiziano Giacomo Allice, Francesco Cerutti, Elena Pomari, Concetta Castilletti, Eleonora Lalle, Fabrizio Maggi, Matteo Fracella, Paolo Ravanini, Giulia Faolotto, Roberta Schiavo, Giuliana Lo Cascio, Carla Acciarri, Stefano Menzo, Fausto Baldanti, Guido Antonelli, Alessandra Pierangeli, Elena Pariani, Antonio Piralla, AMCLI-GLIViRe Working Group, Laura Sandri, Sandro Binda, Federica Giardina, Antonino M G Pitrolo, Patrizia Bono, Gabriele Arcari, Alessandra Lombardi, Antonia Palumbo, Salvatore Curiale, Eva Caterina Borgatti, Federica Tontarelli, Fabrizio Carletti, Ombretta Turriziani, Annamaria Colacicco

**Affiliations:** Laboratory of Clinical Microbiology, Virology and Bioemergencies, Azienda Socio Sanitaria Territoriale Fatebenefratelli Sacco, Milan, Italy; Department of Biomedical Sciences for Health, University of Milan, Milan, Italy; Microbiology and Virology Unit, Fondazione Istituto Ricovero e Cura a Carattere Scientifico Policlinico San Matteo, Pavia, Italy; Department of Specialist and Laboratory Medicine, Unitá Operativa Complessa Microbiology Treviso Hospital, Azienda Unitá Locale Socio Sanitaria 2 La Marca, Treviso, Italy; Department of Specialist and Laboratory Medicine, Unitá Operativa Complessa Microbiology Treviso Hospital, Azienda Unitá Locale Socio Sanitaria 2 La Marca, Treviso, Italy; Microbiology Unit, Istituto Ricovero e Cura a Carattere Scientifico Azienda Ospedaliero-Universitaria di Bologna, Bologna, Italy; Microbiology Unit, Istituto Ricovero e Cura a Carattere Scientifico Azienda Ospedaliero-Universitaria di Bologna, Bologna, Italy; Section of Microbiology, Department of Medical and Surgical Sciences, University of Bologna, Bologna, Italy; Microbiology and Virology Unit, Fondazione Istituto Ricovero e Cura a Carattere Scientifico Ca' Granda Ospedale Maggiore Policlinico, Milan, Italy; Microbiology and Virology Unit, Fondazione Istituto Ricovero e Cura a Carattere Scientifico Ca' Granda Ospedale Maggiore Policlinico, Milan, Italy; Südtiroler Sanitätsbetrieb Laboratory of Microbiology and Virology, Provincial Hospital of Bolzano (Azienda Sanitaria Dell' Alto Adige), Lehrkrankenhaus der Paracelsus Medizinischen Privatuniversität, Bolzano, Italy; Südtiroler Sanitätsbetrieb Laboratory of Microbiology and Virology, Provincial Hospital of Bolzano (Azienda Sanitaria Dell' Alto Adige), Lehrkrankenhaus der Paracelsus Medizinischen Privatuniversität, Bolzano, Italy; Microbiology and Virology Unit, Fondazione Istituto Ricovero e Cura a Carattere Scientifico Policlinico San Matteo, Pavia, Italy; Department of Biomedical Sciences for Health, University of Milan, Milan, Italy; Hygiene Unit, Azienda Ospedaliero-Universitaria Policlinico di Bari, Bari, Italy; Hygiene Unit, Azienda Ospedaliero-Universitaria Policlinico di Bari, Bari, Italy; Hygiene Section, Department of Interdisciplinary Medicine, University of Bari A. Moro, Bari, Italy; Unitá Operativa Complessa Microbiology and Virology, Cotugno Hospital Azienda Ospedaliera di Rilievo Nazionale dei Colli, Naples, Italy; Unitá Operativa Complessa Microbiology and Virology, Cotugno Hospital Azienda Ospedaliera di Rilievo Nazionale dei Colli, Naples, Italy; Laboratory of Clinical Microbiology, Virology and Bioemergencies, Azienda Socio Sanitaria Territoriale Fatebenefratelli Sacco, Milan, Italy; Department of Medicine and Innovation Technology (DIMIT), University of Insubria, Varese, Italy; Laboratory of Medical Microbiology and Virology, University Hospital, Azienda Socio Sanitaria Territoriale Sette Laghi, Varese, Italy; Department of Medicine and Innovation Technology (DIMIT), University of Insubria, Varese, Italy; Laboratory of Medical Microbiology and Virology, University Hospital, Azienda Socio Sanitaria Territoriale Sette Laghi, Varese, Italy; Laboratory of Microbiology and Virology, Amedeo di Savoia Hospital, Azienda Sanitaria Locale Città di Torino, Torino, Italy; Laboratory of Microbiology and Virology, Amedeo di Savoia Hospital, Azienda Sanitaria Locale Città di Torino, Torino, Italy; Department of Infectious-Tropical Diseases and Microbiology, Istituto Ricovero e Cura a Carattere Scientifico Sacro Cuore Don Calabria Hospital, Negrar di Valpolicella, Verona, Italy; Department of Infectious-Tropical Diseases and Microbiology, Istituto Ricovero e Cura a Carattere Scientifico Sacro Cuore Don Calabria Hospital, Negrar di Valpolicella, Verona, Italy; Laboratory of Virology, National Institute for Infectious Diseases Lazzaro Spallanzani Istituto Ricovero e Cura a Carattere Scientifico, Rome, Italy; Laboratory of Virology, National Institute for Infectious Diseases Lazzaro Spallanzani Istituto Ricovero e Cura a Carattere Scientifico, Rome, Italy; Laboratory of Microbiology and Virology, Department of Molecular Medicine, University of Rome La Sapienza, Rome, Italy; Laboratory of Microbiology and Virology, Maggiore della Carità Hospital, Novara, Italy; Laboratory of Microbiology and Virology, Maggiore della Carità Hospital, Novara, Italy; Microbiology and Virology Unit, Hospital Guglielmo da Saliceto Hospital, Piacenza, Italy; Microbiology and Virology Unit, Hospital Guglielmo da Saliceto Hospital, Piacenza, Italy; Department of Medicine and Surgery, University of Parma, Parma, Italy; Department of Biomedical Sciences and Public Health, Polytechnic University of Marche, Ancona, Italy; Department of Biomedical Sciences and Public Health, Polytechnic University of Marche, Ancona, Italy; Virology Unit, Azienda Ospedaliero Universitaria delle Marche, Ancona, Italy; Microbiology and Virology Unit, Fondazione Istituto Ricovero e Cura a Carattere Scientifico Policlinico San Matteo, Pavia, Italy; Department of Clinical, Surgical, Diagnostic, and Pediatric Sciences, University of Pavia, Pavia, Italy; Laboratory of Microbiology and Virology, Department of Molecular Medicine, University of Rome La Sapienza, Rome, Italy; Microbiology and Virology Unit, University Hospital Policlinico Umberto I, University of Rome La Sapienza, Rome, Italy; Laboratory of Microbiology and Virology, Department of Molecular Medicine, University of Rome La Sapienza, Rome, Italy; Department of Biomedical Sciences for Health, University of Milan, Milan, Italy; Microbiology and Virology Unit, Fondazione Istituto Ricovero e Cura a Carattere Scientifico Policlinico San Matteo, Pavia, Italy

**Keywords:** human metapneumovirus, molecular epidemiology, respiratory infections, adults, phylogenetic analysis

## Abstract

**Background:**

Human metapneumovirus (hMPV) infections have a significant impact on public health. However, the extent of this burden in Italy remains poorly defined due to a lack of comprehensive data. The aim of this cross-sectional multicenter study was to understand the epidemiology of hMPV in Italy, particularly in older adults.

**Methods:**

We analyzed laboratory data from molecular respiratory viral diagnostic tests conducted at 17 centers across Italy from September 2022 to August 2024. Respiratory viruses were tested from outpatients for epidemiologic surveillance and from patients presenting to tertiary hospitals for diagnostic purpose. G gene sequencing was performed on a limited number of circulating strains.

**Results:**

Data from 96 460 tests yielded an overall hMPV positivity rate of 3.4%; the hMPV positivity rate was 2.6% in adults aged 50 years and older, a third of whom were aged >80 years. In north-west Italy, hMPV was detected more frequently in outpatients than in hospitalized patients. The temporal distribution of cases showed seasonal peaks in February 2023 and April 2024, which exhibited some geographic variation but overlapped in the general population and in the elderly. Phylogenetic analysis suggested an even distribution of hMPV-A and -B, with a predominance of clades A2c with a 111-nucleotide duplication and B2b, and the possible extinction of previously circulating clades A2c with a 180-nucleotide duplication and B2a.

**Conclusions:**

hMPV was shown to be a relevant respiratory pathogen in older adults, who could be more likely to have severe outcomes. These findings may inform hMPV surveillance and the development of prevention strategies.

Acute respiratory infections (ARI) are a significant public health challenge that will persist beyond the severe acute respiratory syndrome coronavirus 2 (SARS-CoV-2) pandemic [1]. Many different respiratory viruses cause ARI and ARI-related hospitalizations in all age groups. Although SARS-CoV-2, influenza, and respiratory syncytial virus (RSV) have historically been described to cause significant morbidity and mortality [2], human metapneumovirus (hMPV) has also been reported to be associated with a wide variety of clinical manifestations. These range from asymptomatic or mild illness to severe lower respiratory tract disease, in both pediatric and adult populations [3–5]. In addition, like other respiratory viruses, hMPV-associated severe disease is not uncommon in older adults and those with comorbidities [6], who represent a priority target population for hMPV vaccination.

hMPV, an enveloped, negative-stranded RNA virus, is classified into 2 antigenically distinct types, A and B, which are further classified into 4 subtypes (or genotypes), A1, A2, B1, and B2 [7], and lineages. The genetic lineages have been classified based on the variable outer G glycoprotein. Because different nomenclatures are used, efforts are being made to establish a unified classification [8]. Over the course of epidemic seasons, the prevalence of different subtypes and lineages can vary widely [8].

As with other respiratory viruses, the coronavirus disease 2019 (COVID-19) pandemic restrictions resulted in the cessation of hMPV circulation for more than a year [9], creating the so-called immune debt, that is, the waning of general population immunity and the formation of groups of hMPV-naive children [9, 10]. The immune debt may also be responsible for the changes in seasonality observed following the resurgence of hMPV infection after social distancing restrictions were lifted [10].

As respiratory virus circulation returns to usual patterns, national and regional data on hMPV epidemiology and genetic evolution are needed to assess the impact of potential hMPV vaccines.

The aim of this study was therefore to characterize the postpandemic epidemiologic impact of hMPV in Italy by aggregating multicenter virologic laboratory data from all molecular respiratory viral diagnostic tests from September 2022 to August 2024. In addition, several hMPV strains were characterized by phylogenetic analysis based on sequences of the external glycoprotein (G) gene.

## METHODS

**Figure 1.**
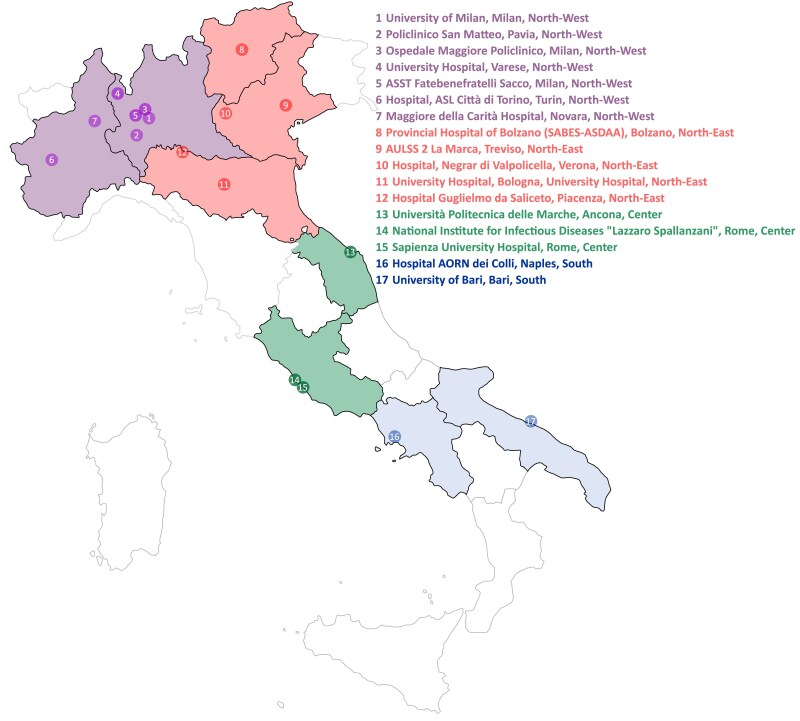
Geographical distribution of the GLIViRe (Working Group on Respiratory Viral Infections) clinical microbiology laboratories participating in the study.

We analyzed data from all molecular respiratory viral diagnostic tests performed between September 2022 and August 2024 in the microbiology and virology laboratories of the Italian Working Group on Respiratory Viral Infections (GLIViRe) throughout Italy. GLIViRe is a network of microbiology laboratories established in November 2018 [11], and includes tertiary and research institutes, as well as academic laboratories (Figure 1).

An email invitation was sent to all GLIViRe laboratories, requesting their participation in a study on hMPV. Data on the total number of respiratory specimens tested and diagnostic results for hMPV were provided by 17 GLIViRe centers throughout Italy (Table 1 and Figure 1). These are academic centers (university hospitals) and tertiary care hospitals, including hospitals accredited as research institutions and large territorial diagnostic centers (Supplementary Table 1).

**Table 1.** hMPV Data From the 17 GLIViRe Laboratories for the Total Study Population and Subjects Aged 50 Years and Older

Laboratory ID No.	Patients’ Setting	Overall Study Population		Subjects Aged 50 y and Older	
		No. Positive/No. Tested	% Positive (95% CI)	No. Positive/No. Tested	% Positive (95% CI)
1	Outpatients	404/6812	5.93 (5.39–6.51)	81/1366	5.93 (4.79–7.31)
2	Inpatients	87/6334	1.37 (1.12–1.69)	50/3973	1.25 (.96–1.66)
	Outpatients	109/3189	3.42 (2.84–4.11)	5/434	1.15 (.49–2.67)
3	Inpatients	433/11 505	3.76 (3.43–4.13)	114/5862	1.94 (1.62–2.33)
4	Inpatients	144/3289	4.38 (3.73–5.13)	39/1586	2.46 (1.8–3.34)
5	Inpatients	179/3537	5.06 (4.37–5.83)	34/1296	2.62 (1.88–3.64)
6	Inpatients	121/3147	3.84 (3.23–4.57)	49/1906	2.57 (1.95–3.37)
7	Both	63/1320	4.77 (3.75–6.06)	16/473	3.38 (2.09–5.42)
Total north-west		1540/39 133	3.94 (3.75–4.13)	388/16 896	2.30 (2.08–2.53)
8	Both	355/10 614	3.34 (3.02–3.70)	133/4909	2.71 (2.29–3.20)
9	Inpatients	388/14 100	2.75 (2.5–3.04)	271/10 851	2.50 (2.22–2.80)
10	Inpatients	111/2548	4.35 (3.63–5.22)	32/854	3.70 (2.63–5.18)
	Outpatients	0/19	NA	0/11	NA
11	Inpatients	392/11 634	3.37 (3.05–3.71)	103/4301	2.39 (1.96–2.90)
	Outpatients	34/399	8.52 (5.97–11.70)	9/132	6.82 (3.16–12.55)
12	Inpatients	44/1263	3.48 (2.60–4.64)	12/541	2.22 (1.27–3.84)
	Outpatients	0/10	NA	0	NA
Total north-east		1324/40 587	3.26 (3.09–3.44)	560/21 599	2.59 (2.40–2.81)
13	Inpatients	18/304	5.92 (3.79–9.16)	1/40	2.50 (.44–12.9)
	Outpatients	0/7	NA	0	NA
14	Inpatients	60/2388	2.51 (1.96–3.22)	37/1569	2.36 (1.72–3.23)
15	Inpatients	52/1622	3.21 (2.45–4.18)	6/465	1.29 (.59–2.79)
Total center		130/4321	3.01 (2.54–3.56)	44/2074	2.12 (1.58–2.84)
16	Both	66/5930	1.11 (.88–1.41)	41/NK	NA
17	Inpatients	170/5000	3.40 (2.93–3.93)	35/796	4.40 (3.18–6.05)
	Outpatients	50/1489	3.35 (2.56–4.4)	10/465	2.15 (1.17–3.91)
Total south		286/12 419	2.30 (2.05–2.58)	86/NK	NA
Total Italy		3280/96 460	3.40 (3.29–3.52)	1068/41 365	2.58 (2.43–2.74)

For each center, the number of inpatients and outpatients tested, if known, or the total number of samples tested, from September 2022 to August 2024 are reported. Inpatients are cases in the hospital setting, including both patients tested in the emergency department and those tested in the hospital wards, and outpatients are cases of influenza-like illness tested in the centers participating in the national surveillance of respiratory viruses (RespiVirNet) [12].

Abbreviations: CI, confidence interval; hMPV, human metapneumovirus; NA, not applicable; NK, not known; GLIViRe, Working Group on Respiratory Viral Infections.

Laboratories extracted data by week from diagnostic records using the following queries: (1) requests for hMPV detection in respiratory specimens (nasopharyngeal swabs, aspirates, bronchoalveolar lavages) collected from patients with symptoms of influenza-like illness (ILI) or ARI without age restriction; (2) requests for hMPV detection in respiratory specimens from patients ≥50 years with ILI or ARI; and (3) requests submitted between 29 August 2022 (week 35, 2022) and 1 September 2024 (week 34, 2024). Duplicate respiratory samples from the same patient were excluded from the analysis if collected less than 14 days apart.

In the hospital setting, patients from the emergency department or from hospital wards (referred to as inpatients) who had been clinically diagnosed with ILI or ARI were tested for respiratory pathogens at the request of the attending physician. Furthermore, several laboratories also performed molecular diagnosis of hMPV in respiratory samples from patients with ILI symptoms who were not hospitalized (referred to as outpatients) (Table 1). These latter centers participated in the national surveillance of respiratory viruses (RespiVirNet) [12].

Molecular testing for respiratory viruses during all months of the study period was performed in 15 laboratories using commercial platforms (Supplementary Table 1), namely: Allplex Respiratory Panel (Seegene) (laboratory Nos. 3, 4, 5, 7, 8, 9, 16, 17); Biofire FilmArray (Biomerieux) (laboratory Nos. 6, 8, 10, 12, 14, 16); FTD21plus (Siemens) (laboratory No. 13); QIAstat-Dx Respiratory SARS-CoV-2 Panel (Qiagen) (laboratory Nos. 14, 15); Allplex RV Essential Assay (Seegene) (laboratory No. 11); and Bosphore Respiratory Pathogens Panel Kit (Anatolia Geneworks) (laboratory No. 10). Two laboratories (Nos. 1 and 2) used laboratory-developed real-time PCR (Supplementary Table 1).

Patients' demographic data were also extracted from diagnostic records and anonymized in accordance with confidentiality requirements. The study was approved by the Ethics Committee of Rome University Hospital (Protocol 0966/2023) and, due to its retrospective nature, informed consent was not required.

### Phylogenetic Analysis

A random selection of residual diagnostic specimens obtained from outpatients and inpatients ≥ 50 years of age at the University of Milan, Pavia and Rome (laboratory Nos. 1, 2, and 15) were subjected to hMPV partial genetic characterization. The entire G gene was amplified [11] and subjected to Sanger sequencing. In addition, 3 Italian hMPV-A 2019 strains identified in a previous study [11], 133 hMPV-A, and 91 hMPV-B sequences from GenBank (https://www.ncbi.nlm.nih.gov/nucleotide/) were added to the datasets (Supplementary Table 2).

### Statistical Analysis

The number of hMPV detections per week and per laboratory were reported. The hMPV detection rate per macrogeographic area and age group was expressed as a crude proportion, with the corresponding 95% confidence interval (CI) with the Wilson interval, assuming a normal distribution. The statistical significance of the observed differences between the proportions in the various groups was determined using Fisher exact test. For continuous variables, Student *t* test was used. Statistical analysis was conducted using the open-source epidemiological statistics software OpenEpi, version 3.03 [13]. The following definitions were used to describe epidemiologic characteristics of epidemic waves: (1) peak value was defined as the maximum number of hMPV-positive specimens out of the total number of samples tested in a given week during the epidemic period; (2) peak time was defined as the week in which the peak was reached; and (3) hMPV epidemic onset was defined as the time when the number of hMPV-positive samples out of the total number of samples exceeded a certain threshold. The threshold was defined as the average number of hMPV-positive samples observed during the study period plus 2 standard deviations, as originally proposed by the US Centers for Disease Control and Prevention [14], and also described by Tabataba et al [15]. A *P* value less than .05 was considered statistically significant (2-tailed test).

## RESULTS

### HMPV Epidemiology

The 17 participating laboratories reported results for 96 460 samples collected from individuals with respiratory illness (ILI or ARI). A total of 3280 of 96 460 respiratory samples tested positive for hMPV in 2 consecutive years (from September 2022 to August 2024), resulting in an overall positivity rate of 3.4%. The positivity rate ranged from 2.3% (286/12 419) in southern Italy to 3.9% (1540/39 133) in the north-western macroarea (Table 1).

As shown in Table 1, most centers tested only inpatients attending the hospital or a limited number of outpatients, and 1 center tested only samples from outpatients with ILI in the framework of the RespiVirNet surveillance. A comparison of hMPV positivity rates among inpatients and outpatients was conducted exclusively for the north-west area, where a substantial number of outpatients were tested (Table 1). The hMPV positivity rates were significantly (*P* < .0001) higher among outpatients (513/10 001; 5.1%) than among inpatients (964/27 812; 3.5%).

### Epidemiology of HMPV in the Population Aged 50 Years and Older

The epidemiology of hMPV infection from samples in individuals aged ≥50 years, representing 42.9% (41 365/96 460) of all samples tested for hMPV, was characterized. Overall, 91% of respiratory samples tested were obtained from the upper respiratory tract. The hMPV-positivity rate in adults aged ≥50 years was 2.6% (1068/41 365), of whom 556/1068 (52.3%) were female. hMPV positivity showed a regional variation, ranging from 2.1% (44/2074) in central Italy to 4.4% (35/796) in southern Italy (Table 1). Comparison of hMPV rates among outpatients and inpatients aged 50 years and older tested in the north-west region (Table 1) showed higher positivity rates among outpatients (86/1800, 4.8% vs 286/14 623, 1.9%; *P* < .0001).

The risk of hMPV positivity was lower in subjects aged ≥50 years (odds ratio [OR], 0.6; 95% CI, .59–.69) than in subjects aged birth to 49 years. Exact age was not available for 5 (0.4%) subjects ≥50 years. The median age of the 1063 hMPV-positive subjects ≥50 years was 74 years (interquartile range [IQR], 64–84 years). Of note, 196/1063 (18.4%) were aged 50–60 years, 231/1063 (21.7%) were aged 61–70 years, 281/1063 (26.3%) were aged 71–80 years, and 355/1063 (33.2%) were aged >80 years. hMPV-positive subjects were significantly older in north-east (median age, 78 years; IQR, 68–86 years) and central Italy (median age, 74 years; IQR, 68–80 years) compared to subjects from the north-west (median age, 70 years; IQR, 60–80 years) and south Italy (median age, 69 years; IQR, 63–81 years) (*P* < .001).

### Temporal Distribution of hMPV Detections

The positivity rates remained consistent between the 2 time periods (2022–2023 and 2023–2024). The hMPV positivity rate was 3.7% and 4.1% in the north-west in 2022–2023 and 2023–2024, respectively. In the north-east, the hMPV-positivity rate was 3.3% in both periods. The hMPV positivity rate was 3.3% and 2.7% in the central area, and 2.7% and 2.0% in the south, during the 2 periods, respectively.

The hMPV positivity rates among adults aged ≥50 years in the Italian macroareas were analyzed in more detail and compared between the study periods, as shown in Table 2. In the north-west, hMPV positivity rates were similar between 2022–2023 and 2023–2024 and during the weeks of the 2 seasonal peaks. In the north-east, hMPV showed trends towards higher positivity rates in 2023–2024 (*P* = .16 and *P* = .03, in season and in peak weeks, respectively). In the central macroarea, a decrease in the hMPV rate was observed in 2023–2024 compared to 2022–2023, which was also observed in the south. However, in the south, hMPV cases were more numerous in the 2023–2024 peak weeks compared to the 2022–2023 peak weeks (Table 2).

**Table 2.** hMPV Positivity Rate in Subjects Aged 50 Years and Older During September 2022 to August 2023 and September 2023 to August 2024 and During the Weeks of Seasonal Peak, in the 4 Italian Macroareas

Macroarea	HMPV Positivity Rates in Subjects ≥ 50 y			HMPV Positivity Rates in Subjects ≥ 50 y During the Seasonal Peak^a^		
	September 2022 to August 2023	September 2023 to August 2024	*P* Value	September 2022 to August 2023	September 2023 to August 2024	*P* Value
North-west	150/6704 (2.24)	238/10 028 (2.37)	.56	wk 5–6, 2023 21/293 (7.2)	wk 13–14, 2024 33/402 (8.2)	.34
North-east	225/9239 (2.44)	334/12 172 (2.74)	.16	wk 9–10, 2023 41/447 (9.2)	wk 15–16, 2024 63/501 (12.6)	.03
Center	26/841 (3.09)	18/1200 (1.48)	.015	wk 3–4, 2023 5/54 (9.3)	wk 17–18, 2024 4/48 (8.3)	.43
South	16/230 (6.96)	19/557 (3.41)	.036	wk 5–6, 2023 4/18 (22.2)	wk 15–16, 2024 5/12 (41.6)	.04

Data are No. positive/No. tested (% positive).

Abbreviations: hMPV, human metapneumovirus; wk, week.

^a^The calendar weeks corresponding to the hMPV peak of cases in the 2 study periods in the 4 Italian macroareas are indicated.

**Figure 2.**
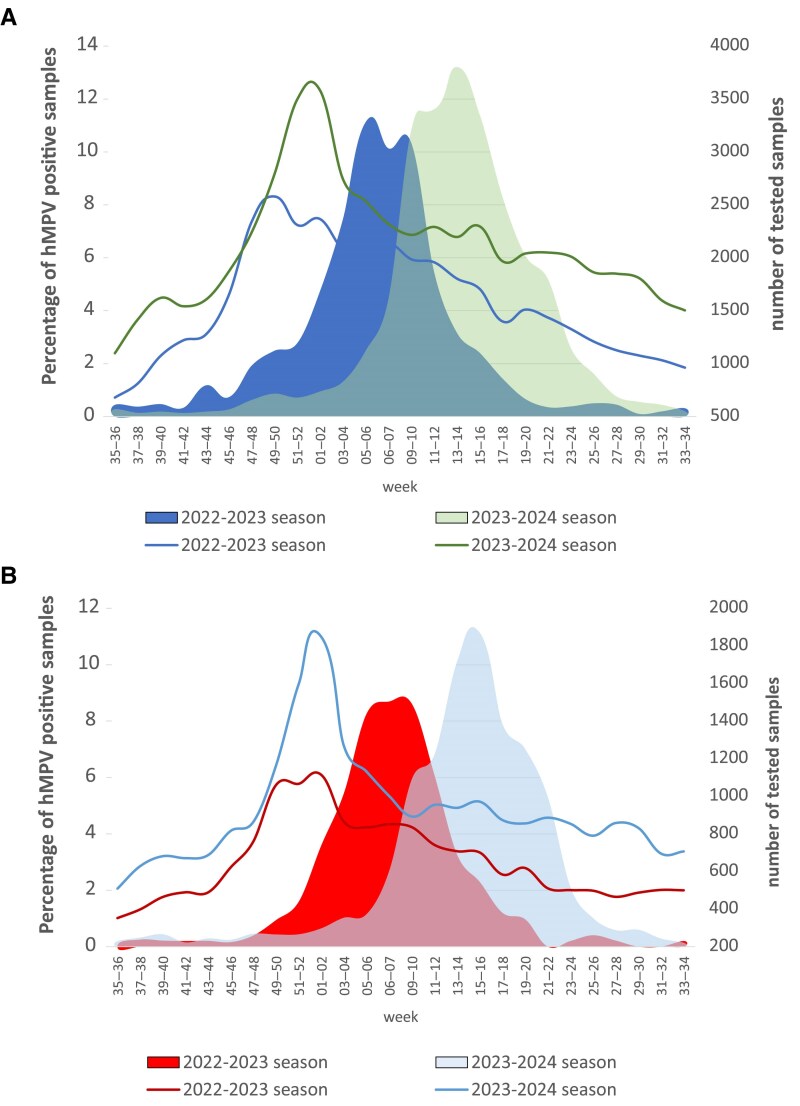
*A*, Total number of respiratory samples tested for human metapneumovirus (hMPV) and hMPV positivity rate on a biweekly basis from week 35, 2022 to week 35, 2024, in Italy. *B*, Number of respiratory samples from subjects aged 50 years and older tested for hMPV and hMPV positivity rate on a biweekly basis from week 35, 2022 to week 35, 2024. The calendar weeks of the study period (September to August) are plotted on the X-axis; the percentage of hMPV-positive cases is plotted on the left Y-axis and represented as differently colored areas for the 2 seasons as shown in the color legend; the total number of respiratory samples tested is plotted on the right Y-axis and represented as differently colored lines as shown in the color legend.

As shown in Figure 2*A*, the temporal distribution of hMPV-positive cases in the total population at the national level revealed the presence of 2 peaks. The first wave started in week 2, 2023, peaked in week 6, 2023 and ended in week 12, 2023, with a duration of 11 weeks. The second wave started in week 8, 2024, peaked in week 13, 2024, and ended in week 20, 2024, with a duration of 13 weeks. The overall hMPV positivity rate at peak was 11.9% in 2022–2023 and 12.8% in 2023–2024. Notably, the peak of hMPV detection observed in 2023–2024 was delayed by 7 weeks compared to the peak observed in 2022–2023.

**Figure 3.**
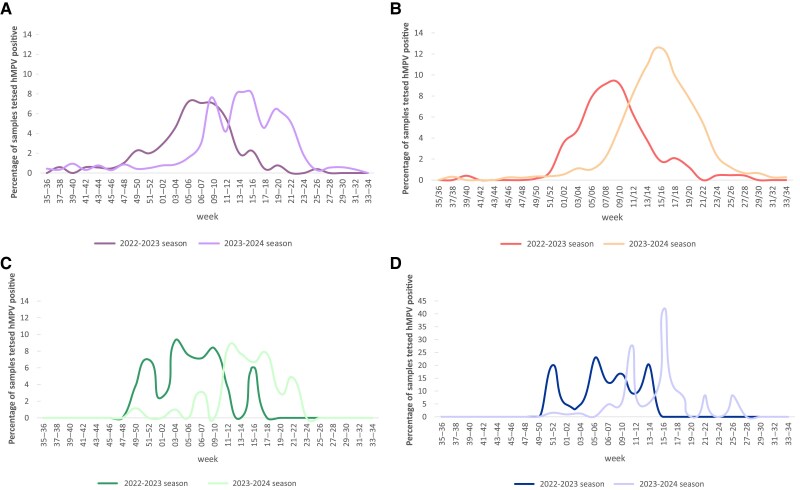
Number of respiratory samples from subjects aged 50 years and older tested for human metapneumovirus (hMPV) and hMPV positivity rate on a biweekly basis from week 35, 2022 to week 35, 2024 stratified by the 4 Italian macroareas: north-west (*A*), north-east (*B*), center (*C*), and south (*D*).

The biweekly distribution of total respiratory specimens and hMPV positivity rate in subjects ≥ 50 years of age, shown in Figure 2*B*, is comparable to that observed in the overall population (Figure 2*A*). As in the total population, the hMPV epidemic curve in adults aged ≥50 years peaked in the 2023–2024 season between 6 and 14 weeks after the peak in the previous season (Figure 2*B*) and varied slightly by region (Table 2 and Figure 3).

### Phylogeny of hMPV Lineages

**Figure 4.**
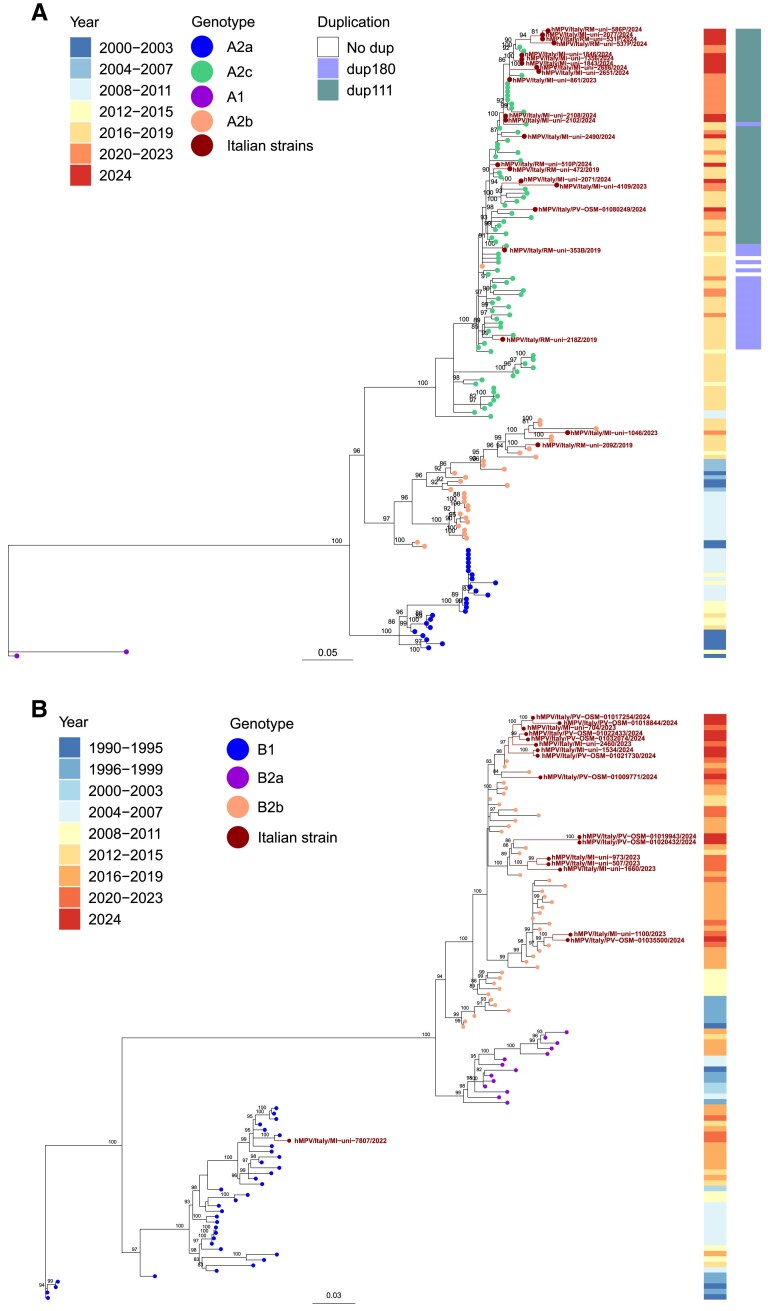
Phylogenetic analysis of the G gene of hMPV strains circulating in Italy. *A*, The phylogenetic tree of hMPV-A includes 18 GLIViRe sequences from study samples (September 2022 to August 2024), 3 GLIViRe sequences from a previous study [11], and 133 reference strains. *B*, The phylogenetic tree of hMPV-B includes 17 GLIViRe sequences from study samples (September 2022 to August 2024) and 91 reference strains. The hMPV-A dataset (155 sequences) was aligned using the strains NL/00/1 (GenBank accession number AF371337) and NL/00/17 (GenBank accession number FJ168779) as references for genotype A1 and A2, respectively. The hMPV-B dataset (108 sequences) was aligned using NL/99/1 (GenBank accession number AY525843) and NL/94/1 (GenBank accession number FJ168778) as reference strains for sublineages B1 and B2, respectively. The alignment tool used was MAFFT version 7.525 [16] and the visualization was performed using MEGA 11 [17]. A maximum likelihood phylogenetic tree of the dataset was constructed using IQ-TREE multicore version 2.3.3 [18] with a nucleotide substitution model identified by ModelFinder [19]. Branch robustness was assessed using the Shimodaira–Hasegawa approximate likelihood-ratio test [20] and ultrafast bootstrap approximation tests [21]. The phylogenetic tree was visualized using the ggtree package [22] and a custom R script [23]. Abbreviations: G, glycoprotein; GLIViRe, Working Group on Respiratory Viral Infections; hMPV, human metapneumovirus

A phylogenetic analysis was performed on 35 sequences of the entire G glycoprotein gene generated by 3 centers located in the north-west (Milan and Pavia) and central (Rome) Italy. Of these, 18 belonged to subtype A and 17 to subtype B. Phylogenetic analysis showed that only 1 hMPV-A strain was of the A2b genotype, while all others were of the A2c genotype (Figure 4*A*). Because the A2c strains carrying duplications of 111 and 180 nucleotides (A2c-dup111 and A2c-dup180) have become predominant worldwide [24, 25], a duplication calling analysis was performed. All study strains of the A2c genotype showed the 111-nucleotide duplication. Phylogenetic analysis of Italian hMPV-B showed that 1 strain was of the B1 genotype, while 16 belonged to the B2b genotype (Figure 4*B*).

## DISCUSSION

HMPV is an important pathogen responsible for upper and lower respiratory tract infections in children and adults worldwide [4–6]. However, there is a lack of documentation on the seasonal prevalence of hMPV and its impact on ILI and ARI cases in Italy, especially in older adults. In the present study, we report the distribution of hMPV cases over a 2-year period, including the 2 respiratory virus seasons following the complete lifting of pandemic restrictions in Italy. This is based on the results of routine molecular testing of over 96 000 respiratory samples.

The data showed that hMPV was initially detected in both the general population and older adults in all study centers in late 2022 and then showed 2 waves during the study period. At the national level, hMPV positivity rates were similar between the 2 periods (September to August 2022–2023 and 2023–2024), with some regional variation. The number of hMPV-positive cases and the positivity rate were higher in the 2023–2024 season than in the previous season in northern Italy, but opposite trends were observed in central and southern Italy. Because cases of ILI or ARI were tested at the request of the attending physician, it is possible that clinical judgment about the need for respiratory multipanel testing differed between hospitals and influenced hMPV positivity rates in the macroareas, particularly where fewer hospitals were included (central and southern Italy). No national or regional algorithms or guidelines have yet been implemented to guide clinicians in respiratory virus testing decisions.

The overall hMPV detection rate of 3.4% is considerably higher than that observed in 2019, in a multicenter study conducted in 8 GLIViRe centers using the same multipanel molecular assays, with the exception of the academic center in Rome [11]. In 2019, hMPV was identified in 2% of samples tested, with rates ranging from 1.2% to 4%; unfortunately, this earlier study did not provide specific hMPV rates in adults aged 50 years and older [11].

Although lower than in younger subjects, the hMPV positivity found in this study in adults ≥50 years of age (2.6%) is noteworthy because in these subjects, the oldest age group was overrepresented, with one-third of cases occurring in subjects over 80 years of age. This observation confirms the results of our previous multicenter study [11], which showed that 30% of all hMPV-positive cases were identified in older adults (≥65 years). Furthermore, the 2.6% hMPV positivity in adults ≥50 years of age is comparable to that found in a recent study on hMPV-positive hospitalized adults (2.8%) [26].

It can be hypothesized that the observed increase in hMPV positivity rates in the postpandemic period, especially in younger age groups, may be due to increased clinician demand for the use of multipathogen panels for the diagnosis of respiratory infections. Another possible factor contributing to increased hMPV circulation in the postpandemic period could be the hMPV-specific immune debt created by pandemic restrictions, that is, a decline in virus-specific population immunity, particularly relevant in children [10].

In our study, the peak of hMPV activity differed between the 2 seasons: in the first, it was recorded in February, whereas in the 2023–2024 season, hMPV showed a peak of cases in April, similar to that observed in north-central Italy before the pandemic [11]. Prior to COVID-19, hMPV epidemics in central Europe alternated between winter and spring-summer every 2 years [27, 28]. In addition, a global analysis showed that annual hMPV epidemics in temperate climates occurred in late winter and spring and usually followed the end of RSV epidemics with a delay of 1.7 months [29]. Indeed, perturbations in hMPV seasonality observed in the postpandemic period [10, 30, 31] have also been attributed to the immune debt that would have favored out-of-season spikes in several viral infections after the end of pandemic restrictions [9]. In this postpandemic context, we observed that the temporal distribution of the hMPV cases showed the earlier seasonal peak in winter when the virus returned to circulation in 2022–2023, followed by a second epidemic with a more typical seasonality in 2023–2024; this distribution is similar to the trend observed in Italy for RSV and influenza [12, 32]. In particular, after the relaxation of restrictions in Italy in the summer of 2021, RSV reemerged and caused an intense epidemic in Italy in the fall of 2021, earlier than its historical trend, while RSV circulation was also intense in the fall-winter of 2022–2023, but peaked in January, more similar to the historical seasonality [32]. Influenza A virus returned to abundant circulation in the 2022–2023 season, with the peak of cases in fall-winter, earlier than registered in Italy in most influenza seasons; the 2023–2024 season was also intense, but with the more usual winter peak [12]. In the absence of historical data for hMPV in Italy, it is not possible to say whether the pandemic has altered the hMPV-specific population immunity, resulting in an earlier peak of hMPV cases in the 2022–2023 season, or whether hMPV winter waves have occurred in the past, alternating with spring waves in other seasons. A recent global analysis used routine surveillance data to assess how the timing of several respiratory viruses changed after the SARS-CoV-2 pandemic [33]. Although not statistically significant due to the paucity of national data, the timing of hMPV peak in the Northern Hemisphere appears to be earlier in the 2022–2023 season compared to prepandemic historical data [33]. Extended surveillance for hMPV is needed to understand whether the temporal pattern and peak intensity observed in the postpandemic period may continue to change over time.

Sequencing and phylogenetic analysis of the highly variable G gene was performed on a representative subset of hMPV-positive samples from north-western and central Italy to clarify whether novel strains were circulating after the pandemic period. No strains belonging to the A1 lineage were identified among hMPV-A cases in the present study or in the prepandemic period in Italy [11], consistent with previous reports indicating that no A1 strains have been detected worldwide since 2006 [8, 31]. The A2b lineage was represented by a single case in 2023, while the A2c lineage was predominant, with only A2c-dup111 strains identified. The A2c-dup111 and A2c-dup180 variants have been in circulation since 2012, gradually replacing the previously dominant A2a and A2b lineages [8, 31]. The duplication in G would confer an evolutionary advantage on both strains with the duplication by masking the conserved antigenic epitopes of the F glycoprotein [31], but A2c-dup111 has become dominant compared to A2c-dup180. Indeed, no A2c-dup180 has been detected in the Netherlands since 2019 [31], while in Italy the strain with the longer insertion still accounted for 25% of A2c cases in 2019 [11]. This phylogenetic analysis confirms the prepandemic trend of A2c-dup111 dominance, which may have been accelerated by pandemic restrictions causing the disappearance of A2c-dup180 strain in Italy. However, given the limited number of cases that have been sequenced, it is possible that strains that were not detected after the pandemic are still circulating in areas where surveillance is not in place.

Among hMPV-B cases, 1 genotype B1 was identified during the 2022–2023 season, consistent with low detection rates in Italy in 2019 [11]. In the prepandemic period, genotype B1 represented a minority of hMPV cases worldwide [8, 31], with the exception of an outbreak in Asian countries in 2017 [34]. As for the B2 genotype, clade 2a, found in northern Italy in 2019 [11], it was not identified in this study; in contrast, clade B2b was the dominant hMPV-B clade in the postpandemic seasons. The B2b clade is thought to have originated from the B1 genotype and spread mainly between 2012 and 2021 [31], with a notable increase in prevalence between 2012 and 2015 (Figure 4*B*). Several strains of the B2b clade have undergone an evolutionary process and appear to be dominant in the postpandemic period (Figure 4*B*). In contrast to B2b, the monophyletic B2a clade, which was predominantly circulating before 2004, was not detected after the onset of the SARS-CoV-2 pandemic (Figure 4*B*).

The global postpandemic molecular epidemiology of hMPV has been characterized in only a few studies. In the Netherlands, hMPV-A clade A2.2.2 carrying the 111-nucleotide duplication in G (also known as A2c-dup111) was predominant in 2021 [8]. In Taiwan, A2c-dup111, which was dominant in 2021, was not detected in 2022 while the B2 genotype was dominant in 2023 [35]. In Korea, the hMPV 2022 cases were A2.2.1 (the alternative name for A2b) and B2 strains [27]. In Australia, an A2b variant emerged in 2019 and became dominant in 2020–2021; this variant cocirculated with B1 and B2 genotypes in 2021 [10]. In Japan, hMPV-B B1 and B2, but not hMPV-A, were identified following the SARS-CoV-2 pandemic [36]. These data suggest that the pandemic restrictions may have caused distinct local events of predominance and extinction of hMPV genetic lineages, which could potentially lead to further genetic divergence and new evolutionary events. Further surveillance studies using whole-genome sequencing should determine the potential for hMPV genetic variants to spread to distant geographic locations and generate unexpected outbreaks [31, 34].

This study has several limitations. First, the study design is limited to 2 years, which may affect the generalizability of the epidemiologic results, and the study is retrospective in nature, which precludes the inclusion of clinical information. In particular, subjects tested in the emergency department are considered here as inpatients because the vast majority were subsequently hospitalized in wards or intensive care units, although the exact number is not known. In addition, it was not possible to obtain detailed age data for all patients tested for hMPV, only for hMPV-positive adults aged 50 years and older, and we could not calculate any age bias in testing. Second, the GLIViRe centers used different molecular methods, laboratory-developed real-time PCR or European union in vitro diagnostic device directive-compliant multiplex assays, which may have different analytical sensitivities. However, 8 and 6 laboratories, respectively, used the same commercial platform for hMPV detection (Allplex Respiratory Panel and Biofire FilmArray), which is likely to reduce analytical variability and increase confidence in estimating hMPV positivity. Third, the number of hMPV-positive samples and the genomic region sequenced are relatively limited. This is due to the limited availability of residual diagnostic specimens and to the different laboratory capacity in terms of sequencing. To address the latter issue, the variability in terms of different sequencing machines was considered in the bioinformatics analysis.

Nevertheless, this study represents a significant contribution to the knowledge of hMPV in Italy, demonstrating the national and regional rates of this infection in the elderly population. Epidemiologic analysis of hMPV circulation in the postpandemic seasons provides insight into potential shifts in hMPV epidemiology. In addition, phylogenetic analysis of circulating strains suggests potential changes in hMPV lineages in the postpandemic era. These findings may prove valuable in the differential surveillance and management of hMPV disease and in the development of prevention strategies.

## Supplementary Material

jiaf111_Supplementary_Data

## Supplementary Data

Supplementary materials are available at *The Journal of Infectious Diseases* online (http://jid.oxfordjournals.org/). Supplementary materials consist of data provided by the author that are published to benefit the reader. The posted materials are not copyedited. The contents of all supplementary data are the sole responsibility of the authors. Questions or messages regarding errors should be addressed to the author.

## Notes

***Members of the AMCLI GLIViRe working group.*** Laura Sandri, Sandro Binda (Department of Biomedical Sciences for Health, University of Milan, Milan); Federica Giardina (Department of Clinical, Surgical, Diagnostic and Pediatric Sciences, University of Pavia, Pavia, Italy); Antonino M. G. Pitrolo (Microbiology and Virology Unit, Fondazione IRCCS Policlinico San Matteo, Pavia, Italy); Patrizia Bono (Microbiology and Virology Unit, Fondazione IRCCS Ca' Granda Ospedale Maggiore Policlinico, Milan); Gabriele Arcari (Laboratory of Medical Microbiology and Virology University Hospital, ASST Sette Laghi, Varese); Alessandra Lombardi (Laboratory of Clinical Microbiology, Virology and Bioemergencies, ASST Fatebenefratelli Sacco, Milan); Antonia Palumbo (Laboratory of Microbiology and Virology, Maggiore della Carità Hospital, Novara); Salvatore Curiale (Department of Infectious-Tropical Diseases and Microbiology, IRCCS Sacro Cuore Don Calabria Hospital, Negrar di Valpolicella, Verona); Eva Caterina Borgatti (Section of Microbiology, Department of Medical and Surgical Sciences, University of Bologna, Bologna); Federica Tontarelli (Virology Unity, AOU delle Marche, Department of Biomedical Sciences and Public Health, Università Politecnica delle Marche, Ancona); Fabrizio Carletti (Laboratory of Virology, National Institute for Infectious Diseases “Lazzaro Spallanzani” IRCCS, Rome); Ombretta Turriziani, (Laboratory of Microbiology and Virology, Department of Molecular Medicine, University of Rome La Sapienza, Rome); Annamaria Colacicco (Virology Laboratory-Microbiology and Virology Unit, University of Bari-Policlinico of Bari, Bari).

***Author contributions.*** A. M. and L. P. contributed to the study methodology and conducted data analysis. G. R. contributed to the study methodology and performed phylogenetic analysis. V. B., G. P., A. C., E. M., C. G., F. Cen., E. F., V. M., F. N., F. Cer., E. Po., and E. L. performed sample collection and molecular diagnosis. E. V., T. L., S. U. R., E. Pag., C. C., F. M., M. C., C. T., N. M., T. A., P. R., G. Fa., R. S., G. L. C., C. A., and S. M. collected patients’ demographic data and performed data analysis. G. Fe. and M. F. performed data analysis, and prepared tables and figures. F. B. and G. A. contributed to the study methodology, funding acquisition, and reviewed and edited the manuscript. Al. P., E. Par., and An. P. designed the study, supervised data analysis and wrote the original draft of the manuscript. Members of the AMCLI GLIViRe working group extracted diagnostic data. All the authors read and approved the final manuscript.

***Data availability***. The sequence data generated in this study have been submitted to GenBank under the accession numbers PQ609627–PQ609665. The diagnostic data are available from the corresponding author upon reasonable request. Accession numbers of the sequences used as reference in this study are reported in Supplementary Table 2.

***Financial support.*** This work was supported by the European Union within the Next Generation European Union-Ministero Universitá e Ricerca Piano Nazionale Ripresa e Resilienza (PNRR) extended partnership initiative on emerging infectious diseases (grant number PE00000007, One Health Basic and Translational Research Actions addressing Unmet Needs on Emerging Infectious Diseases INF-ACT).

***Supplement sponsorship***. This article appears as part of the supplement “Human Metapneumovirus Disease Burden in Older Adults and Implications for Adult Vaccination Strategies,” sponsored by AstraZeneca.
