# Supplementary material for: Multicenter Cross-sectional Study on the Epidemiology of Human Metapneumovirus in Italy, 2022–2024, With a Focus on Adults Over 50 Years of Age
Source: J Infect Dis. 2025 Jul 16;232(Suppl 1):S109–20. doi: 10.1093/infdis/jiaf111 (PMC12265061; doi:10.1093/infdis/jiaf111)
Supplement: jiaf111_Supplementary_Data [file jiaf111_supplementary_data.zip › Pierangeli_TableS1 REV.docx]

**Table S1.** Affiliation and hMPV detection method of the 17 GLIViRe centers. The microbiology laboratories of the GLIViRe network are academic centers (A) i.e. university hospitals, or tertiary care hospitals (T), including hospitals accredited as research institutions and large territorial diagnostic centers; their geographic locations are shown in Figure 1.

| **Lab. No.**  **(Type)** | **Affiliation** | **hMPV detection method** |
| --- | --- | --- |
| 1 (A) | Department of Biomedical Sciences for Health, University of Milan, Milan | Home-made^a^ |
| 2 (A) | Microbiology and Virology Unit, Fondazione IRCCS Policlinico San Matteo, Pavia | Home-made^b^ |
| 3 (T) | Microbiology and Virology Unit, Fondazione IRCCS Ca' Granda Ospedale Maggiore Policlinico, Milan | Allplex^TM^ Respiratory Panel (Seegene) |
| 4 (A) | Laboratory of Medical Microbiology and Virology University Hospital, ASST Sette Laghi Varese | Allplex^TM^ Respiratory Panel Seegene) |
| 5 (A) | Laboratory of Clinical Microbiology, Virology and Bioemergencies, ASST Fatebenefratelli Sacco, Milan | Allplex^TM^ Respiratory Panel (Seegene) |
| 6 (T) | Laboratory of Microbiology and Virology, Amedeo di Savoia Hospital, ASL Città di Torino, Turin | Filmarray Respiratory 2.1 and Pneumonia Plus panels (Biomerieux) |
| 7 (T) | Laboratory of Microbiology and Virology, Maggiore della Carità Hospital, Novara | Allplex^TM^ Respiratory Panel (Seegene) |
| North-West | | |
| 8 (T) | Laboratory of Microbiology and Virology, Provincial Hospital (SABES-ASDAA), Bolzano | Allplex^TM^ Respiratory Panel (Seegene), Filmarray Respiratory 2.1 panel (Biomerieux) |
| 9 (A) | UOC Microbiology Treviso Hospital, Department of Specialist and Laboratory Medicine, AULSS 2 La Marca, Treviso | Allplex^TM^ Respiratory Panel (Seegene) |
| 10 (T) | Department of Infectious-Tropical Diseases and Microbiology, IRCCS Sacro Cuore Don Calabria | Filmarray Respiratory 2.1 panel (Biomerieux), Bosphore Respiratory Pathogens Panel Kit (Anatolia Geneworks) |
| 11 (A) | Microbiology Unit, IRCCS Azienda Ospedaliero-Universitaria di Bologna, Bologna | AllplexTM RV Essential Assay (Seegene) |
| 12 (T) | Microbiology Unit, Hospital Guglielmo da Saliceto, Piacenza | Filmarray Respiratory 2.1 panel (Biomerieux) |
| North-East | | |
| 13 (A) | Virology Unity, AOU delle Marche/Department of Biomedical Sciences and Public Health, Università Politecnica delle Marche, Ancona | FTD Respiratory Pathogens 21 (Siemens-Healthineers) |
| 14 (T) | Laboratory of Virology, National Institute for Infectious Diseases "Lazzaro Spallanzani", Rome | QIAstat-Dx® Respiratory SARS-CoV-2 Panel (Qiagen), Filmarray Respiratory 2.1 panel (Biomerieux) |
| 15 (A) | Microbiology and Virology Laboratory, Sapienza University Hospital, Rome | QIAstat-Dx® Respiratory SARS-CoV-2 Panel (Qiagen) |
| Center | | |
| 16 (T) | UOC Microbiology and Virology, Cotugno Hospital AORN dei Colli, Naples | Allplex^TM^ Respiratory Panel (Seegene), Filmarray Respiratory 2.1 panel (Biomerieux) |
| 17 (A) | Virology Laboratory - Microbiology and Virology Unit - University of Bari - Policlinico of Bari, Bari | Allplex^TM^ Respiratory Panel (Seegene) |
| South | | |

^a^ Home-made method described in ref 11: Pierangeli A, Piralla A, Uceda Renteria S, et al. Multicenter epidemiological investigation and genetic characterization of respiratory syncytial virus and metapneumovirus infections in the pre-pandemic 2018-2019 season in northern and central Italy. Clin Exp Med. 2023 ; 23 :2725-37.

^b^ Home-made assay performed using QuantiFast®Pathogen RT-PCR+IC Kit (Qiagen) with the following primers and probe: MPV-for 5’-tayatggagatgagcaaaactccy-3’, MPV-rev 5’-ggaccatgytyactgcacttgatt-3’, MPV-probe FAM-aatgacycttcatatgccytgcaag-MGB, and cycling conditions: 50 °C for 20 minutes, 95 °C for 5 minutes, 45 cycles at 95 °C for 15 seconds and 60 °C for 30 seconds.
