# Supplementary material for: Multicenter Cross-sectional Study on the Epidemiology of Human Metapneumovirus in Italy, 2022–2024, With a Focus on Adults Over 50 Years of Age
Source: J Infect Dis. 2025 Jul 16;232(Suppl 1):S109–20. doi: 10.1093/infdis/jiaf111 (PMC12265061; doi:10.1093/infdis/jiaf111)
Supplement: jiaf111_Supplementary_Data [file jiaf111_supplementary_data.zip › Supplementary material S2.docx]

**Table S2.** Reference sequences used for phylogenetic analyses

| **NCBI accession number** | | | |
| --- | --- | --- | --- |
| **RSV-A** | | **RSV-B** | |
| A2a | FJ168779.1 | B2b | HM197719.1 |
| A2a | KC403979.1 | B2b | KC403972.1 |
| A2a | KC403981.1 | B2b | KC562222.1 |
| A2a | KC403982.1 | B2b | KC562227.1 |
| A2a | KF686742.1 | B2b | KC562229.1 |
| A2a | KJ627388.1 | B2b | KC562238.1 |
| A2a | KJ627394.1 | B2b | KC562239.1 |
| A2a | KJ627390.1 | B2b | KJ627397.1 |
| A2a | KJ627398.1 | B2b | KJ627414.1 |
| A2a | KJ627407.1 | B2b | KJ627432.1 |
| A2a | KJ627413.1 | B2b | KX829147.1 |
| A2a | KJ627415.1 | B2b | MF045425.1 |
| A2a | KJ627416.1 | B2b | MG431250.1 |
| A2a | KJ627418.1 | B2b | MK588637.1 |
| A2a | KJ627419.1 | B2b | MN617494.1 |
| A2a | KJ627421.1 | B2b | MN617522.1 |
| A2a | KJ627423.1 | B2b | MN617545.1 |
| A2a | KJ627425.1 | B2b | MN617549.1 |
| A2a | KJ627427.1 | B2b | MN617553.1 |
| A2a | KJ627433.1 | B2b | MN617563.1 |
| A2a | KX829083.1 | B2b | OM243133.1 |
| A2a | KY474534.1 | B2b | OM243138.1 |
| A2a | MK588635.1 | B2b | OM243140.1 |
| A2a | MN617420.1 | B2b | OM243210.1 |
| A2a | MN617500.1 | B2b | OM243313.1 |
| A2c | MN617453.1 | B2b | OM243321.1 |
| A2c | MN617445.1 | B2b | OM243324.1 |
| A2c | MN617488.1 | B2b | OM243329.1 |
| A2c | MN617560.1 | B2b | OM243349.1 |
| A2c | GQ153651.1 | B2b | OM243389.1 |
| A2c | KX829070.1 | B2b | OM243393.1 |
| A2c | KX829074.1 | B2b | OM243399.1 |
| A2c | KY474531.1 | B2b | OM243412.1 |
| A2c | KY474535.1 | B2b | OM243432.1 |
| A2c | KY474539.1 | B2b | OM243537.1 |
| A2c | KY474541.1 | B2b | OM243541.1 |
| A2c | KY474542.1 | B2b | OM243557.1 |
| A2c | KY474544.1 | B2b | OM243569.1 |
| A2c | MN617443.1 | B2b | OM243571.1 |
| A2c | MN617451.1 | B2b | OM243579.1 |
| A2c | MN617491.1 | B2b | OM243630.1 |
| A2c | MN617568.1 | B2b | OM243632.1 |
| A2c | MN867464.1 | B2a | AY297748.1 |
| A1 | KC562226.1 | B2a | DQ843658.1 |
| A1 | KU821121.1 | B2a | JN184402.1 |
| A2b | MN617428.1 | B2a | KC562228.1 |
| A2b | OM243187.1 | B2a | KC562231.1 |
| A2b | DQ843659.1 | B2a | KC562232.1 |
| A2b | KC403978.1 | B2a | KC562237.1 |
| A2b | KC403983.1 | B2a | KF530178.1 |
| A2b | KC403984.1 | B2a | MN617404.1 |
| A2b | KC562221.1 | B2a | MN617461.1 |
| A2b | KC562224.1 | B2a | MN617558.1 |
| A2b | KC562225.1 | B2a | OM243334.1 |
| A2b | KC562233.1 | B2a | OM243134.1 |
| A2b | KC562240.1 | B2a | EF535506.1 |
| A2b | KC562243.1 | B1 | AY525843.1 |
| A2b | KJ627379.1 | B1 | JN184401.1 |
| A2b | KJ627385.1 | B1 | KC403974.1 |
| A2b | KJ627382.1 | B1 | KC562219.1 |
| A2b | KJ627392.1 | B1 | KC562223.1 |
| A2b | KJ627401.1 | B1 | KC562234.1 |
| A2b | KJ627402.1 | B1 | KC562242.1 |
| A2b | KJ627405.1 | B1 | KC562230.1 |
| A2b | KJ627412.1 | B1 | KC562235.1 |
| A2b | KJ627417.1 | B1 | KF530163.1 |
| A2b | KJ627420.1 | B1 | KF530164.1 |
| A2b | KJ627422.1 | B1 | KF530167.1 |
| A2b | KJ627430.1 | B1 | KF530171.1 |
| A2b | KX829092.1 | B1 | KF530173.1 |
| A2b | KY474529.1 | B1 | KF530179.1 |
| A2b | KY474530.1 | B1 | KJ627383.1 |
| A2b | KY474537.1 | B1 | KJ627431.1 |
| A2b | MN617472.1 | B1 | KJ627435.1 |
| A2b | OM243288.1 | B1 | KF516922.1 |
| A2c | MN745084.1 | B1 | KX829134.1 |
| A2c | MN745085.1 | B1 | KX829144.1 |
| A2c | MN745086.1 | B1 | KX829162.1 |
| A2c | MN745087.1 | B1 | MK588636.1 |
| A2c | MN617460.1 | B1 | MN306019.1 |
| A2c | MN617503.1 | B1 | MN617413.1 |
| A2c | MN617535.1 | B1 | MN617473.1 |
| A2c | MN617546.1 | B1 | MN617489.1 |
| A2c | OM243139.1 | B1 | MN617505.1 |
| A2c | OM243202.1 | B1 | MN617515.1 |
| A2c | OM243304.1 | B1 | OM243365.1 |
| A2c | OM243352.1 | B1 | OM243416.1 |
| A2c | OM243368.1 | B1 | OM243455.1 |
| A2c | OM243404.1 | B1 | OM243490.1 |
| A2c | OM243422.1 | B1 | OM243536.1 |
| A2c | OM243428.1 | B1 | OM243549.1 |
| A2c | OM243484.1 |  |  |
| A2c | OM243568.1 |  |  |
| A2c | OM243580.1 |  |  |
| A2c | OM243620.1 |  |  |
| A2c | OM243621.1 |  |  |
| A2c | OM243626.1 |  |  |
| A2c | OM243629.1 |  |  |
| A2c | OM243648.1 |  |  |
| A2c | OM243651.1 |  |  |
| A2c | OM243653.1 |  |  |
| A2c | OM243655.1 |  |  |
| A2c | OM243665.1 |  |  |
| A2c | OM243670.1 |  |  |
| A2c | OM243672.1 |  |  |
| A2c | OM243676.1 |  |  |
| A2c | OM243682.1 |  |  |
| A2c | OM243683.1 |  |  |
| A2c | OM243805.1 |  |  |
| A2c | OM243385.1 |  |  |
| A2c | KX829081.1 |  |  |
| A2c | KX829113.1 |  |  |
| A2c | MN617441.1 |  |  |
| A2c | MN617487.1 |  |  |
| A2c | MN617504.1 |  |  |
| A2c | MN617510.1 |  |  |
| A2c | MN617525.1 |  |  |
| A2c | MN617536.1 |  |  |
| A2c | MN617564.1 |  |  |
| A2c | OM243146.1 |  |  |
| A2c | OM243177.1 |  |  |
| A2c | OM243212.1 |  |  |
| A2c | OM243216.1 |  |  |
| A2c | OM243280.1 |  |  |
| A2c | OM243330.1 |  |  |
| A2c | OM243450.1 |  |  |
| A2c | OM243473.1 |  |  |
| A2c | OM243523.1 |  |  |
| A2c | OM243567.1 |  |  |
| A2c | OM243602.1 |  |  |
| A2c | OM243609.1 |  |  |
| A2c | OM243644.1 |  |  |
| A2b | KX829112.1 |  |  |
